# Why Only Efficiency, and Not Efficacy, Matters in Psychotherapy Practice

**DOI:** 10.3389/fpsyg.2021.603211

**Published:** 2021-05-07

**Authors:** Henrik Berg

**Affiliations:** ^1^Centre for the Study of the Sciences and the Humanities, Faculty of Humanities, University of Bergen, Bergen, Norway; ^2^Centre for Sustainable Healthcare Education, Faculty of Medicine, University of Oslo, Oslo, Norway

**Keywords:** effectiveness and efficiency, critique, psychotherapy practice and research, evidence-based practice in psychology, facts and values

## Abstract

Evidence-based practice in psychology consists of two quality parameters. One of these quality parameters is efficacy and the other is efficiency. In this article, it is argued that the only relevant parameter for determining quality in clinical care is efficiency. Moreover, emphasising efficacy in psychotherapy practice is symptomatic of a scientocentric ideal in evidence-based practice in psychology. The proper understanding and use of scientific findings entail leaving this scientocentric ideal. In addition, it is crucial that efficiency is related to the ethical aims that constitutes psychotherapy practice.

## Introduction

Cochrane’s (1999) “*Effectiveness and efficiency”* set new standards for medical practice. He argued that without the guidance of randomised controlled trials, medical doctors delivered suboptimal, and sometimes even harmful, services. Because randomised controlled trials were believed to be bias-free, and consequently did not share the biases that led medical doctors to provide suboptimal treatment, Cochrane (1999) wanted randomised controlled trials to be the building blocks of a modernised health-care system. Randomisation aims at providing (a minimum of) two similar groups that are tested in controlled experimental conditions. Any difference in outcome is attributed to the intervention which is normally given to one of the groups only.

Cochrane used the term “effectiveness” to denote the scientific merits of a given treatment. Cochrane recognises that he uses the term effectiveness to denote what is commonly coined “efficacy” (which he avoids simply because he dislikes the word). More specifically, effectiveness refers to the causal effects of a treatment. A treatment is deemed “effective” if scientific findings indicates that it causes a given change in the health status of its recipients. According to Cochrane, randomised controlled trials are superior in bringing about information of causal factors. Due to their lack of bias, randomised controlled trials let researchers draw unequivocal inferences (of whether a treatment is effective or not). Thus, there is an implicit methodological hierarchy in Cochrane’s ideal for clinical science, but Cochrane’s ideal for science and practice are not identical.

There is an often-overlooked political ambition of Cochrane’s thinking, which was to provide effective health-care treatment available for everyone. This political aim led him to coin a second term. Efficiency includes factors such as the economic cost of various treatment options and the optimal use of staff and equipment. Efficiency is the translation of scientific research in clinical setting, taking the most important external (i.e., extra-scientific) factors into consideration. By distinguishing effectiveness and efficiency, Cochrane underscored that clinical science differs from clinical practice. Whereas the models of science constructively reduce complexity, the reduction of complexity in clinical practice is associated with the risk of suboptimal clinical care.

Another important distinction in Cochrane’s thinking is that between cure and care. Cochrane used the term cure to denote the combination of effectiveness and efficiency. One reason for combining effectiveness and efficiency in a concept is to highlight that effective treatments have variable efficiency in different contexts. Thus, one could either identify more effective cures or change the clinical setting to increase efficiency. He also introduced the term care, which are the affectionate aspects of patient treatment (in his own words, “tender” and “loving”) (Cochrane, 1999). While recognising that care is important in actual health-care practice, Cochrane’s recipe for better health-care services was improving cure, particularly emphasising effectiveness. The vision that Cochrane presented in “Effectiveness and efficiency” has led to a major restructuring of health-care systems world-wide (Timmermans and Berg, 2003; Claridge and Fabian, 2005; Shah and Chung, 2009; Sur and Dahm, 2011).

## Evidence-Based Medicine

The legacy of Cochrane prevails in evidence-based medicine and other evidence-based health-care practices. The effectiveness-efficiency distinction exists (albeit in new forms) in most evidence-based medicine models (Sackett, 1997). However, there are some notable differences between Cochrane’s thinking and evidence-based medicine. Whereas Cochrane (1999) insisted that randomised controlled trials are necessary to provide best treatment, contemporary evidence-based medicine’s conceptualisation of best evidence is more flexible (Guyatt and Rennie, 2002). These models have an evidence-based hierarchy ranging randomised controlled trials (and compilations of randomised controlled trials in systematic reviews and meta-analyses) as the most stringent types of evidence. However, through the GRADE-system, research studies are evaluated and ranked from “high” to “very low.” Accordingly, randomised controlled trials with biases can have a ranking of “low” or even “very low” and observational studies can have a ranking of “moderate” or “high.” Although the GRADE-system determine treatment efficacy, it is also relevant for the understanding of effectiveness. Once it is recognised that different kinds of methodologies, and not only randomised controlled trials, can provide useful insight, it opens up for the active interpretation of evidence. The latter versions of evidence-based medicine emphasise the clinical importance of a clinical expert and de-emphasise the direct clinical importance of randomised controlled trials (Haynes et al., 2002).

In contrast to Cochrane’s model, the more recent evidence-based medicine models are tripartite and consists of best external evidence, clinical expertise and patient values (Guyatt, 1991; The Evidence-Based Medicine Working Group, 1992; Sackett et al., 1996; Sackett, 1997; Haynes et al., 2002). This is a fairly important development, because it complicates the notion of efficiency. In accordance with Cochrane, the clinical expert is deemed necessary to convert the best external evidence into best clinical practice. Whereas science consists of propositions typically describing effects, clinical experts translate the scientific findings in a given clinical context. Second, patient values must be included in treatment. This sharpens the distinction between efficacy and efficiency, because patient preferences might diverge from the evidence base. According to this ideal, a treatment can hardly be called efficient, if its effects are not welcomed by the patient. Thus, in the tripartite evidence-based medicine models, efficiency entails individualised treatments.

## Evidence-Based Practice in Psychology

Evidence-based medicine was the template for the American Psychological Association’s policy statement for evidence-based practice in psychology (Levant, 2005). The authors of the policy-statement declares that [e]vidence-based practice in psychology is […] consistent with the past 20 years of work in evidence-based medicine (Levant, 2005, p. 271). Evidence-based practice in psychology is defined as: “the integration of the best available research with clinical expertise in the context of patient characteristics, culture, and preferences” (American Psychological Association, 2006, p. 273). However, evidence-based practice in psychology is an unsuccessful attempt to create a tripartite model. It actually exists of only one part, which is best available research. Thus, clinical expertise and patient characteristics, culture and preferences are in fact scientific sub-categories (Berg, 2019). In addition, the integration of these (alleged) three parts have not been substantiated. It is the clinical expert that must integrate the different elements in evidence-based practice. Thus, the competence of the clinician must somehow reflect the three (alleged) parts in evidence-based practice in psychology (Berg, 2020). The consequence of these failings is that evidence-based practice in psychology contains a direct link from scientific evidence to clinical practice. In spite of major short-comings, it continues to be the dominating regulating principle for psychotherapy practice (Levant, 2005).

However, there are also problems with regards to the definition of best evidence, in evidence-based practice in psychology. At first sight, the notion of evidence in the policy statement is quite inclusive. It contains a section with the heading “multiple types of research evidence.” Under this heading it is argued that “[m]ultiple research designs contribute to evidence-based practice, and different research designs are better suited to address different types of questions” (Levant, 2005, p. 274). Some of the research methods mentioned are “clinical observation,” “systematic case-studies,” and “qualitative methods.” These methods typically have a lower standing in evidence-based practice (miscellaneous). In addition, methods such as RCTs and meta-analyses are included (Levant, 2005).

However, another section of the policy-statement describes “specific interventions” (Levant, 2005). In this section, Cochrane’s distinction between effectiveness and efficiency re-emerges (Cochrane, 1999) with the concepts *treatment efficacy* and *clinical utility*:

*Treatment efficacy* is defined as the systematic and scientific evaluation of whether a treatment works. *Clinical utility*, on the other hand, is defined as the applicability, feasibility, and usefulness of the intervention in the local or specific setting where it is to be offered. Clinical utility also includes determination of the generalisability of an intervention whose efficacy has been established (Levant, 2005, p. 275).

In the quality parameter called *treatment efficacy* there is a distinction between three different types of evidence forming an evidence hierarchy. The least reliable kind of evidence is clinical opinion, observation and consensus followed by systematised clinical observation. Randomised controlled experiments [sic] at the top of the hierarchy. The policy statement argues that randomised controlled experiments “represent a more stringent way to evaluate treatment efficacy because they are the most effective way to rule out threats to internal validity in a single experiment” (Levant, 2005, p. 275). This reasoning resonates Cochrane’s stance on randomised controlled trials.

If randomised controlled trials are preferred for evaluating treatment efficacy for specific interventions, the notion of specific intervention in psychotherapy needs clarification. A specific intervention is not the same as a specific technique (e.g., exposure-therapy or behavioural experiment) (Bennet-Levy et al., 2004; Chrétien et al., 2017). If a psychotherapist is intervening to strengthen the affective bond (as a part of the therapeutic alliance) (Horvath and Bedi, 2002; Horvath et al., 2011), the intervention is specific, but it does not necessarily involve a specific technique. If every intervention with a given aim is defined a specific intervention, most therapist interventions are indeed specific. It follows that the evidence-hierarchy applies to every therapeutic action, in the widest sense of the word. Thus, randomised controlled trials are preferred to indicate efficacy. Consequently, randomised controlled trials are as dominant in evidence-based practice in psychology as in evidence-based medicine, only with the disadvantage of being dominant in a somewhat opaque manner.

Cochrane’s distinction between care and cure has been confused by the authors of the policy-statement for evidence-based practice in psychology. The very distinction is obsolete in psychotherapy, because care (e.g., empathy, congruence, affective bond, etc.) is the better part of cure (i.e., what makes psychotherapy work) (Norcross, 2011; Wampold, 2015; Wampold and Imel, 2015). A grim example is the attempt to create a list of “empirically validated/supported psychotherapies” based on a narrow understanding of psychotherapy (Chambless et al., 1993). The same misunderstanding seems to have informed the policy-statement for evidence-based practice in psychology. It is very unclear what the actual role of other research designs is to be in actual practice.

The other quality parameter is *clinical utility.* In the policy-statement it is argued that:

At a minimum this includes attention to generality of effects across varying and diverse patients, therapists, settings, and the interaction of these factors; the robustness of treatments across various modes of delivery; the feasibility with which treatments can be delivered to patients in real-world settings; and the costs associated with treatments (Levant, 2005, p. 275).

Clinical utility denotes the ability to combine different sources of knowledge to find the optimal treatment in real-life settings. In addition, clinical utility includes economic factors. Leaving economic factors aside, it should be clear that that the definition of an optimal real-world treatment is by no means straightforward. There is a large number of fact and value entanglements in psychotherapy practice making the notion of utility equivocal. Clinical practice concerns itself with the unique individual patient. The most relevant question when facing an individual is whether one has the knowledge, skill and resources to help that very individual. If the clinician has knowledge about effects that do not pertain to that very individual, lack the skill to convert knowledge into practice and/or do not have the resources to provide the treatment, knowledge about a given form of treatment is irrelevant. In that sense, knowledge about treatment efficacy qua treatment efficacy is useless for clinical practice. It is useful if and only if it leads to clinical utility. Thus, insofar that evidence-based practice in psychology aims at regulating psychotherapy *practice*, the only relevant parameter is clinical utility.

The move from empirically supported treatments (Chambless and Hollon, 1998; Chambless et al., 1998; Chambless, 1999) to evidence-based practice in psychology was supposed to be a move toward a more inclusive ideal for best practice (Levant, 2004; Peterson, 2004). Nonetheless, evidence-based practice in psychology have remains of the scientism of empirically supported treatments (Chambless et al., 1993, 1998; Chambless and Hollon, 1998; Chambless, 1999). One of these is that scientific findings *per se* serves as quality indicator of clinical practice. However, treatment efficacy is only be a quality indicator for research and not for practice.

The proper understanding of efficiency comes with a major caveat. The sound use of scientific research in psychotherapy practice depends on the conceptualisation of psychotherapy. The propositions of psychotherapy research are value-laden and the various psychotherapy schools and interventions merges with ethics (Tjeltveit, 1999, 2004; Woolfolk, 2015; Berg and Slaattelid, 2017). Psychotherapy schools are constituted by differing normative claims. As an example, the normative aim of existential psychotherapy differs from that of cognitive-behavioural therapy. Any consideration of research findings for clinical practice has to take this facet of psychotherapy into consideration. Empirical research cannot determine the nature of the good. The good and the right are the subjects of normative ethics. Thus, there is an inherent and principal limitation in scientific research when informing psychotherapy practice.

When defining “real-world effects” one has to take into consideration that psychotherapy aims at realising a vision of a better life (Waring, 2016; Berg, 2020). This entails that moral and ethics are quintessential to psychotherapy. High quality science can be very helpful in achieving many of those aims, but it presupposes a sensible conceptualisation of the relationship between science and practice. Thus, all in all, ethics must come first and science come second.

## Conclusion

Because the policy-statement for evidence-based practice in psychology aims at regulating practice through scientific and extra-scientific parameters, it should encompass the difference between science and practice. In the current version of the policy-statement there is a failed attempt to achieve this. This does not only lead to a series of misunderstandings as to what makes psychotherapy “work,” but also with misunderstandings of what psychotherapy *is*. Ultimately, the distance from science (as it is defined in evidence-based practice in psychology) to clinical practice is considerable, because the former deal with empirical regularities at a group level and the latter deal with the realisation of a better life at the level of the individual. Without sorting out these misconstructions, there is little hope that evidence-based practice in psychology could serve as a satisfactory regulatory principle.

## Data Availability Statement

The original contributions presented in the study are included in the article/supplementary material, further inquiries can be directed to the corresponding author/s.

## Author Contributions

The author confirms being the sole contributor of this work and has approved it for publication.

## Conflict of Interest

The author declares that the research was conducted in the absence of any commercial or financial relationships that could be construed as a potential conflict of interest.
